# Guided Internet-Based Cognitive Behavioral Therapy for Adult Depression and Anxiety in Routine Secondary Care: Observational Study

**DOI:** 10.2196/10927

**Published:** 2018-11-28

**Authors:** Kim Mathiasen, Heleen Riper, Tonny E Andersen, Kirsten K Roessler

**Affiliations:** 1 Department of Psychology Faculty of Health Sciences University of Southern Denmark Odense Denmark; 2 Centre for Telepsychiatry Mental Health Services of Southern Denmark Odense Denmark; 3 Department of Mental Health Amsterdam Public Health Research Institute Vrije Universiteit Amsterdam Amsterdam Netherlands; 4 Department of Research and Innovation Specialized Mental Health Care GGZ InGeest Amsterdam Netherlands

**Keywords:** anxiety, cognitive therapy, cohort studies, depression, Internet, secondary care

## Abstract

**Background:**

Internet-based cognitive behavioral therapy (iCBT) is a promising new treatment method for depression and anxiety. However, it is important to determine whether its results can be replicated in routine care before its implementation on a large scale. Although many studies have demonstrated the efficacy of iCBT under controlled conditions, only a few studies have investigated its effectiveness in routine care. Furthermore, several effects of iCBT such as treatment effects in routine care are unclear.

**Objective:**

This study aimed to evaluate the clinical effectiveness of iCBT for depression and anxiety in routine secondary care.

**Methods:**

n a retrospective cohort study, we analysed patients treated for depression or anxiety in a dedicated iCBT clinic in secondary care in Denmark. Patients were examined before treatment and weekly thereafter by using the Patient Health Questionnaire-9 and the Generalized Anxiety Disorder-7 scales for the diagnoses of depression and anxiety, respectively. Primary analyses were conducted using a linear mixed-effects model with random slope and intercept. Secondary analyses were conducted using baseline characteristics as predictors (gender, age, highest level of education, occupational status, marital status, psychotropic medication use, consumption of alcohol, and leisure drugs). Additionally, logistic regression analyses were used to predict noncompletion of treatment.

**Results:**

A total of 203 (depression, N=60; anxiety, N=143) patients were included. Participants were mainly female (78.3% with depression and 65.7% with anxiety), with a mean age of 36.03 (SD 10.97) years (range, 19-67 years) for patients with depression and 36.80 (SD 13.55) years (range, 19-69 years) for patients with anxiety. The completion rates were 62% (37) and 40% (57) for depression and anxiety treatments, respectively. The primary analyses revealed large and significant reductions in the symptom levels of depression (beta=-6.27, SE 0.83, *P*<.001, d=1.0) and anxiety (beta=-3.78, SE 0.43, *P*<.001, d=1.1). High baseline severity of the primary disorder was associated with high treatment gains (r=-0.31 for depression; r=-0.41 for anxiety). In patients with anxiety, high baseline severity also predicted a high risk of noncompletion (odds ratio=1.08, CI=1.01-1.16, *P*=.03). An increase in the baseline severity of the comorbid disorder slightly increased the risk of noncompletion for both disorders (depression: odds ratio=1.03, CI=1.01-1.06, *P*=.02; anxiety: odds ratio=1.08, CI=1.01-1.16, *P*=.03).

**Conclusions:**

iCBT can be clinically effective in routine care. Since depression and anxiety are costly and debilitating disorders that are vastly undertreated, this finding is important. Additionally, iCBT may help bridge the gap between the need for treatment and its provision. Our results are comparable to the within-group results of efficacy and effectiveness studies. Our noncompletion rates are similar to those observed in psychotherapy but are higher than those reported in similar clinics. Multiple factors predicted outcome and noncompletion. However, all predictor effects were statistically weak.

## Introduction

### Background

Anxiety and depression are highly prevalent and costly disorders [1-4]. Although effective treatments for these disorders exist [5], only about half of those in need receive professional help, and only approximately 16% receive minimally adequate evidence-based treatments [6,7]. Many studies have demonstrated the efficacy of guided internet-based cognitive behavioral therapy (iCBT) [8-13], which may help bridge the gap between the need for treatment and its provision.

Although effectiveness studies such as randomized controlled trials (RCTs) conducted in routine care settings [14] are limited in number, they show effect sizes comparable to those obtained under almost-ideal conditions of efficacy studies [15]. Owing to the increasing number of persons seeking help for mental disorders in Denmark, the Danish Agency for Digitisation included iCBT in a national action plan for telemedicine in 2012, which led to the establishment of the first iCBT clinic in Denmark. The clinic, named Internetpsykiatrien, has been in operation since 2014 and a part of routine care since 2015. Funded by the Danish healthcare system, it is free at the point of use.

Internetpsykiatrien is a part of secondary care in the Mental Health Services of Southern Denmark. It delivers guided iCBT [15] for adult patients with anxiety and depression and offers selfreferral; as such, patients can apply for treatment directly at the clinic from a secure and dedicated website, and no general practitioner is required [16]. Herein, we present the results of an observational study of the first routine outcome measurements of a cohort of adult patients who were treated at the clinic with iCBT for depression or anxiety.

This observational cohort study with a pre-post design based on routine outcome measurements is important, as treatments and patients in routine practice may differ from those in RCTs. Patients may differ with regard to their sociodemographic and baseline characteristics as well as their motivation or need for treatment. Additionally, adherence to strict research protocols and treatment manuals and continuous monitoring of clinician adherence are not part of the standard clinical practice in most settings. Thus, although effectiveness trials have higher external validity than efficacy trials, their clinical effect may differ from that in actual routine practice, for example, in the case of RCTs, even in a routine care setting [14,17]. Therefore, studies of the clinical effect of routine care are needed to increase knowledge on the issue in real-world settings [18]. Although efficacy studies are important, the external validity of an intervention should be the prime focus in order to convince decision makers and clinicians of its effectiveness, thereby ensuring its implementation and uptake [19]. In addition to efficacy and effectiveness studies, observational cohort studies can provide important insights into the impact of iCBT in real-life routine care of populations.

The literature on cohort studies in routine practice of comparable iCBT clinics is limited. However, some published studies from the Internetpsykiatrien clinic in Sweden, the eMeistring clinic in Norway, the MindSpot clinic in Australia, and a centralized clinic in Canada [16,20-25] describe dedicated iCBT clinics that deliver guided iCBT and are operated as routine mental health care services. In the Swedish clinic, large, significant within-group effect sizes were found in cohort studies of major depression (n=1203, *d*=1.27) [20], panic disorder (n=570, *d*=0.91) [21], and social anxiety (n=547, *d*=0.86) [22]. In Norway, results for panic disorder showed a large pre-post effect size (n=124, *d*=1.24) [23]. In the Sydney clinic, a cohort study from the first 30 months of operation revealed large, significant effect sizes (n=6149, *d*=1.3-1.4) [24] for transdiagnostic iCBT for anxiety and depression. The centralized unit in Canada offers guided iCBT with selfreferral and provides assistance to community mental health services; the clinic showed large, significant reductions in symptom levels of both anxiety and depression (n=260, *d*=1.2-1.4) [25]. The literature, although scarce, seems to suggest that guided iCBT may be a promising treatment format in routine practice.

### Primary Aim

This study aims to improve knowledge of the clinical effect of guided iCBT in routine practice by presenting and discussing analyses of routine outcome measurements in a cohort of adults treated for anxiety or depression at the iCBT clinic Internetpsykiatrien in Odense, Denmark.

### Secondary Aim

Our secondary aim was to determine predictors of treatment outcome and noncompletion for iCBT.

## Methods

### Design

This uncontrolled retrospective cohort study included all patients who had initiated treatment for depression (N=60) or anxiety (N=143) at the iCBT clinic Internetpsykiatrien and provided consent for analysis of their data. Data for all patients were collected from the time reliable data were available until the time of data extraction (June 1, 2017). For patients with anxiety, reliable data was available from March 1, 2015, and for depressed patients, from August 1, 2016. The study was observational in nature and did not interfere with the normal operation of the clinic. All data were collected as routine outcome measurements and part of continuous quality assurance by the clinic by using online questionnaires. This article adheres to the Strengthening the Reporting of Observational Studies in Epidemiology guidelines for reporting observational studies [26,27] and the Checklist for Reporting Results of Internet E-Surveys (CHERRIES) [28].

### Interventions

Internetpsykiatrien operates as part of routine care in Southern Denmark and is funded by the Region of Southern Denmark, which is a tax-funded public authority. The clinic uses self-referral via a website [29], where patients fill out a secure application form. They do not need referral from other sources such as a general practitioner. Treatment including the use of the programs is free of charge for the patients. After an initial screening of their application, patients are contacted for clarification by telephone and, if appropriate (eg, in cases of increased suicidal risk), provided access to more relevant sources of assistance or invited to a video-based assessment interview with a licensed psychologist or a psychologist under the supervision of a licensed psychologist. Eligibility criteria for patients at the clinic are age ≥ 18 years; meeting the diagnostic criteria of the International Classification of Diseases and Related Health Problems, 10th edition, for major depressive disorder, panic disorder, agoraphobia, social phobia, specific phobia, or generalized anxiety disorder; not currently at high risk of suicide; no comorbid substance dependence, bipolar affective disorder, psychotic illness, or obsessive compulsive disorder; not currently under other psychological treatment for depression or anxiety; access to a personal computer and fast internet connection; and adequate understanding of spoken and written Danish.

After assessment, the patients were provided anxiety or depression treatment. Both treatments included access to an iCBT treatment program and weekly or biweekly clinical support from a licensed clinical psychologist or a psychologist under the supervision of a licensed psychologist. Support was provided via a secure text module for depression treatment and via telephone for anxiety treatment. In addition, support comprised technical assistance, help with interventions included in the programs, and encouragement to continue the treatment. The Internetpsykiatrien has been described in detail elsewhere, and its implementation has been compared to similar clinics internationally [30,31].

For depression treatment, the program NoDep by Context Consulting was used. This program was designed and developed as part of a Public Private Innovation project between the Region of Southern Denmark and Context Consulting when the Internetpsykiatrien was under development. The manuscript was written by Specialist Psychologist Krista Nielsen Straarup, who is a well-known expert in CBT for depression in Denmark. The program includes 6 mandatory modules and 2 optional modules and is based on CBT; its core components include psychoeducation, behavioral activation, cognitive restructuring, and relapse prevention. The optional modules include interventions for reducing rumination and restructuring dysfunctional beliefs [32].

The anxiety treatment uses the Danish version of FearFighter, which is distributed in Denmark by Context Consulting [33] and includes 9 mandatory modules based on CBT. FearFighter was originally developed by Professor Dr. Isaac Marks [34] in England. Its core components comprise psychoeducation, in vivo and interoceptive exposure exercises, cognitive restructuring, and relapse prevention. In both programs, patients are reminded to access the programs by SMS and email on a weekly basis, if they fail to do so. This feature can be switched off by the patient. The programs are accessed via the internet on patients’ own devices. Access link, username, and access codes are provided to the patient by the clinic.

In both programs, clinicians are provided with administrative access, which includes a status of the progress of patients, their exercises, their scores on the weekly measures, and a secure text module (for NoDep only). Further, the clinicians are notified of high scores on suicidal ideation and lack of progress. In both cases, patients are contacted personally by phone. In cases of suicidal risk, a standard procedure including risk assessment and possible referral to an acute ward is instigated.

### Ethics

All the patients analyzed in the study provided informed consent for use of their data at the beginning of treatment. No participants were approached by the research team. The Regional Committees on Health Research Ethics for Southern Denmark waived the need for full approval owing to the retrospective nature of the study, no new intervention was performed, and no human biological material was used. The routine outcome measurement database was approved by the Danish Data Protection Agency.

### Measures

The Patient Health Questionnaire-9 (PHQ-9) [35] was used to evaluate changes in the severity of depressive symptoms. This 9-item questionnaire has good psychometric properties [36,37] and can be used to monitor patients with depressive disorders. All items are scored on a 0- to 3-point scale with a total score of 0-27, and higher scores indicated more severe depression. One study of the Danish version confirmed unidimensionality and reliability but suggests collapsing the two middle response categories [38]. However, the scale used in the Internetpsykiatrien clinic followed the original structure of the questionnaire.

The Generalized Anxiety Disorder-7 (GAD-7) scale [39] was used to measure general symptoms of anxiety. It is a 7-item questionnaire originally developed to measure GAD in primary care; each item is scored 0-3, with a total score of 0-21. This scale has shown good psychometric properties, has been validated in primary care [39] and the general population [40], and has performed well as a measure of anxiety-symptom severity in specialized mental health care settings [41]. Thus far, there are no validation studies of the Danish version of the GAD-7 scale.

The Fear Questionnaire [42] was used to describe baseline levels of comorbid anxiety symptoms for patients with depression. Data were extracted from the questionnaire included in the application form. The Fear Questionnaire measures phobias across 6 subscales and provides a total phobia score of 0-120. Similar to the GAD-7 scale, this scale has demonstrated good psychometric properties [43,44].

All questionnaires were administered in an electronic format in the treatment program or at the initial application form. This was not an open survey, as data were obtained from patients included in treatment at the Internetpsykiatrien clinic. It was mandatory to fill out the questionnaires with no extra incentives, apart from the added value of the supporting psychologist who monitored patients’ progress. Completeness of the questionnaires was checked by the program, such that progress was hindered until all items were completed. Once the questionnaire was submitted, it could not be altered. All responses were unique to the individuals, as its administration was a part of the treatment program or linked to the application form. All participants viewed the questionnaires and participated by completing at least some of the items. All available data were analyzed, even if partly filled questionnaires were submitted.

### Description of Participants

Baseline characteristics of patients were extracted from a questionnaire delivered as part of the online application form, which was used when patients applied for treatment, and from a pretreatment questionnaire package. The collected data on patient characteristics included gender, age, highest level of education, occupational status, marital status, baseline symptomatic levels of depression and anxiety (PHQ-9, GAD-7, Fear Questionnaire), units of alcohol consumed per week, and use of recreational drugs. The baseline characteristics were analyzed using descriptive statistics.

### Primary Analyses

Outcome measures were extracted from the pretreatment questionnaire package and the last of a series of weekly measures administered online as an integrated part of the iCBT programs. The intermittent weekly measures were unfortunately unavailable to the research team.

Treatment effects were estimated using mixed-effects linear regression. The pre-post measurements were considered as repeated observations nested within patients. The slope estimates for the binary treatment variable (0, pre; 1, post) indicated change in the outcome measurements from baseline to follow-up. The primary outcome variables, that is, symptomatic measures of the primary disorder, were included as a fixed effect, whereas the binary treatment variable and subject were included as random effects (random slope and intercept). The slope-intercept correlations were estimated to control for baseline severity. The primary model estimated the overall treatment effect with one slope parameter, one intercept, and one correlation between slope and intercept. No control variables were included. The standardized effect sizes were calculated by taking into account the correlation between the pre-post measurement and expressed as *d* [45]. Multivariate imputation by chained equations was applied to manage missing data. All inferences assumed normally distributed error terms and heteroscedasticity, which were substantiated by visual inspection of the Q-Q normality plot and the plot of fitted values versus standardized residuals.

### Predictor Analyses

Predictors of change in symptom severity were determined in subsequent univariate models; specifically, we employed one model per predictor by using the same model type as that in the primary analyses. Regression estimates for the interaction between the predictor variables and the binary treatment variable indicated the extent to which the overall treatment slope was affected by a particular predictor variable. Additionally, two multivariate models were performed. One model included all variables, and the other model included only significant variables from the univariate analyses.

### Completion

Analyses of treatment effect among completers and non-completers were conducted using the same model type as that of the primary analyses. Logistic regression was used to predict the risk of noncompletion on the basis of all available baseline variables for each of the two patient groups, which was expressed as odds ratios (ORs). Treatment completion was defined as completion of at least 5 of 6 mandatory modules in the depression program and 8 of 9 modules in the anxiety program. These thresholds were applied because patients at these stages of the programs had completed all core active treatment interventions aimed at symptom reduction.

### Statistical Analyses

All data were analyzed using R ver. 3.4.2 [46]. Mixed-effects linear regression was performed using the nlme package [47]. The glm function [46] was used for logistic regression, whereas the mice package was used for imputation [48]. *P*<.05 was set as the threshold value for significance in inferential statistics.

## Results

### Description of Participants

**Figure 1:**
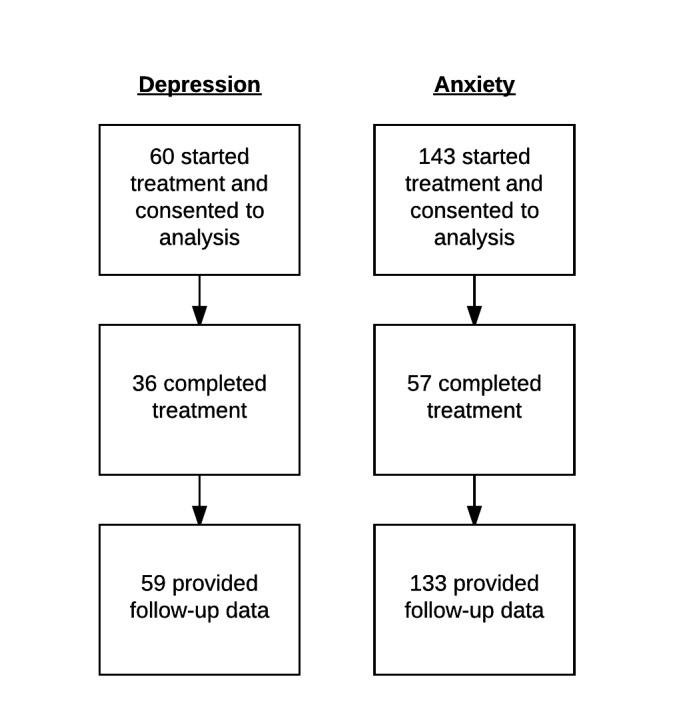
Patient flow chart.

Among patients with depression, 60 patients started treatment and consented to the analysis, of which 59 (98.3%) patients provided followup data. A mean of 5.4 (SD 2.2) of the 6 mandatory and 2 optional modules were completed. In addition, 143 patients with anxiety commenced treatment, of which 133 (93.0%) patients provided followup data. A mean of 5.9 (SD 2.6) modules were (Figure 1). Table 1 presents the characteristics of all the patients who commenced treatment in the inclusion periods and had quit the program or ended treatment by the point of data extraction.

**Table 1:** Description of patients.

Variable		Depression (N=60)	Anxiety (N=143)
**Gender, n (%)**			
	Female	47 (78.3)	94 (65.7)
	Male	13 (21.7)	52 (34.3)
**Age (years)**			
	Mean (SD)	36.03 (10.97)	36.80 (13.55)
	Range	19-67	19-69
**Education, n (%)**		**N=41**	**N=48**
	Primary: 9 years in school	6 (14.6)	6 (12.5)
	Gymnasium: 12 years in school	5 (12.2)	11 (22.9)
	College or vocational school	26 (63.4)	31 (64.6)
	University: 17 years in school	0 (0.0)	0 (0.0)
	Other	4 (9.8)	0 (0.0)
**Occupational status, n (%)**		**N=41**	**N=48**
	Employed/student	23 (56.1)	39 (81.3)
	Unemployed	5 (12.2)	7 (14.6)
	Sick leave	5 (12.2)	2 (4.2)
	Retired	0 (0.0)	0 (0.0)
	Other	8 (19.5)	0 (0.0)
**Marital status, n (%)**		**N=41**	**N=48**
	Single	11 (26.8)^a^	18 (37.5)^b^
	Married or de facto not living together	5 (12.2)^a^	2 (6.25)^b^
	Married or de facto living together	25 (61.0)^a^	27 (56.3)^b^
**Depression severity**			
	PHQ-9^c^ mean (SD)	15.5 (5.7)	9.94 (6.06)
	PHQ-9^a^, range	5-26	0-26
**Anxiety severity**			
	FQ^b^ score, total mean (SD)	22 (22.8)^c^	—^d^
	FQ total score, range	0-81^c^	—
	GAD-7^e^ score, mean (SD)	—	10.42 (5.11)
	GAD-7 score, range	—	0-21
**Patients taking psychotropic medication, n (%)**			**N=136**
	No medication	45 (75.0)	95 (69.9)
	<1 month	1 (1.7)	5 (3.7)
	<2 months	3 (5.0)	2 (1.5)
	>2 months	11 (18.3)	34 (25.0)
**Units of alcohol comsumed weekly, n (%)**			**N=136**
	0	32 (53.3)	60 (44.1)
	0-5	25 (41.7)	51 (37.5)
	5-10	1 (2.7)	17 (12.5)
	10-20	2 (4.1)	7 (5.1)
	>20	0 (0.0)	1 (0.7)
Recreational drugs, n (%)		3 (5.0)	0 (0.0)^f^

^a^PHQ-9: Patient Health Questionnaire-9.

^b^FQ: Fear Questionnaire.

^c^Taken from the application form.

^d^Not applicable.

^e^GAD-7: Generalized Health Anxiety questionnaire.

^f^n=136 because some data were not collected for all patients at the clinic.

### Primary Analyses

Table 2 shows the results of the linear mixed-effects model for depression and anxiety treatments including beta-coefficients, standard errors, and *P*-values for intention-to-treat analyses, completer analysis, and non-completer analysis.

Primary analysis of patients with depression revealed a significant reduction in depressive symptoms on the PHQ-9 (beta=–6.27, SE 0.83, *P*<.001), with a large effect size (*d*=1.0). For patients with anxiety, the linear mixed-effects model demonstrated a significant reduction in the symptom level of anxiety on the GAD-7 (beta=–3.78, SE 0.43, *P*<.001) with a large effect size (*d*=1.1).

### Predictor Analyses

Exploratory predictor analyses for interaction effects revealed that some baseline characteristics predicted a change in symptom severity of the primary disorder from pretreatment to posttreatment (Table 2). For patients with depression, comorbid anxiety predicted slightly lesser symptom reduction with a small, but significant, positive addition to the slope (beta=.08, SE 0.04, *P*=.03). Additionally, a very small, but significant, interaction between treatment and time spent in the program was observed (beta=–0.03, SE 0.01, *P*=.02), indicating that for each additional day spent in the program, the PHQ-9 score reduced by 0.03. Furthermore, a college education as the highest education level predicted a steeper slope from pretreatment to posttreatment on the PHQ-9 (beta=–5.06, SE 2.02, *P*=.02) as compared to primary school education. The baseline symptom level of depression (intercept) was negatively correlated with the slope of change (*r*=–0.31), indicating a larger symptomatic reduction if the baseline level of depressive symptoms was higher. However, when all variables were included in one multivariate model, none of the interactions were significant. Similar results were observed when only the significant interactions from the univariate analyses were included in one multivariate model.

For patients with anxiety, two baseline characteristics predicted outcome. Inclusion in the “Other” category significantly increased symptom reduction (beta=–3.49, SE 1.10, *P*=.002) as compared to inclusion in the “Employed/student” category. Additionally, a higher level of comorbid depressive symptoms at baseline predicted a slightly larger reduction in the symptom level of anxiety (beta=–0.18, SE 0.07, *P*=.01). Time spent in the program was not associated with the treatment outcome in patients with anxiety. Finally, similar to patients with depression, the baseline level of anxiety was negatively correlated with slope (*r*=–0.41), indicating a larger improvement with higher baseline levels. Multivariate analysis of all variables indicated that marriage or de facto not living together was significant (beta=–2.64, SE 1.32, *P*=.047), whereas marriage and de facto living together was not significant (beta=1.63, SE 1.12, *P*=.15). Occupational status included in the “Other” category (beta=–2.96, SE 0.083, *P*=.02) and baseline comorbidity (beta=–0.259, SE 0.083, *P*=.002) remained significant in the multivariate analysis. Similar results were obtained when only the significant interactions from the univariate analyses were included; however, in this case, no category under the variable “marital status” was significant.

### Completion

Of all patients with depression, 37 (61.7%) completed the treatment in the depression program. Analysis of treatment effect showed that for patients with depression, the level of depressive symptoms on the PHQ-9 reduced significantly (beta=–7.41, SE 1.00, *P*<.001), with a large effect size (*d*=1.1). Similarly, noncompleters showed a significant decrease in symptom severity (beta=–4.43, SE 1.39, *P*=.004), albeit with a lower yet moderate-to-large effect size (*d*=0.7). A nonsignificant trend was noted in the interaction between treatment effect and noncompletion (beta=2.97, SE 1.68, *P*=.08), indicating a nonsignificant decrease in symptom change in cases of noncompletion.

Among patients with anxiety, 57 (39.9%) completed treatment. A significant reduction in the severity of anxiety was observed among patients who completed the program (beta=–4.72, SE 0.65, *P*<.001), with a large effect size (*d*=1.1). Similar results were observed for 86 (60.1%) patients who did not complete the program, albeit with a moderate effect size (beta=–3.22, SE 0.56, *P*<.001, *d*=0.6). Furthermore, no significant difference was observed in the interaction between severity of anxiety and noncompletion (beta=1.49, SE 0.88, *P*=.09), but a trend toward better outcome with completion was noted.

**Table 2:** Univariate linear mixed-effects model outcomes for severity of depression and anxiety symptoms. Baseline severity correlated with the main effect for depression (r=–0.31) and anxiety (r=–0.41).

Variables				Depression (PHQ-9)^a^, N=60			Anxiety (GAD-7)^b^, N=143		
				Mean beta (SE)	*P* value^c^	*d*	Mean beta (SE)	*P* value^c^	*d*
**Treatment effect^d^**									
	Main effect			–6.27 (0.83)	<.001	1.0	–3.78 (0.43)	<.001	1.1
	Completers			–7.41 (1.00)	<.001	1.1	–4.72 (0.65)	<.001	1.1
	Noncompleters			–4.43 (1.39)	.004	0.7	–3.22 (0.56)	<.001	0.6
**Predicting outcome^e^**									
	**Gender**								
		Time point		–4.77 (1.79)	.01		–3.90 (0.73)	<.001	
		Gender x time point		–1.91 (2.02)	.35		0.19 (0.91)	.84	
	**Age**								
		Time point		–3.89 (2.88)	.18		–3.77 (1.25)	.003	
		Age x time point		–0.07 (0.08)	.39		0.00 (0.03)	.99	
	**Highest education level**								
		Time point		–3.75 (1.73)	.03		–3.00 (1.54)	.05	
		**Education level x time point**							
			Gymnasium vs primary	1.08 (2.99)	.72		0.28 (1.76)	.88	
			College vs primary	–5.06 (2.02)	.02		–0.50 (1.69)	.77	
			University vs primary				–2.07 (2.03)	.31	
			Other vs primary	0.45 (2.56)	.86		–2.21 (1.81)	.22	
	**Marital status**								
		Time point		–4.86 (1.36)	<.001		–4.33 (0.76)	<.001	
		**Marital status x time point**							
			Married or de facto not living together vs single	1.46 (2.39)	.55		–0.95 (1.09)	.39	
			Married or de facto living together vs single	–3.42 (1.78)	.06		1.94 (1.00)	.05	
	**Occupational status**								
		Time point		–6.46 (1.24)	<.001		–2.54 (0.71)	<.001	
		**Income x time point**							
			Unemployed vs employed or student	1.46 (2.62)	.58		–1.20 (1.07)	.27	
			Sick leave vs employed or student	1.21 (2.26)	.59		–0.46 (1.32)	.73	
			Other vs employed or student	–1.20 (2.26)	.60		–3.49 (1.10)	.002	
	**Baseline comorbidity**								
		**Anxiety (FQ^e^ total)**							
			Time point	–8.16 (1.18)	<.001				
			Time point x FQ total	0.08 (0.04)	.03				
		**Depression (PHQ-9)**							
			Time point				–2.02 (0.81)	.01	
			Time point x PHQ-9				–0.18 (0.07)	.01	
	**Time spent in the program**								
		Time point		–3.95 (1.26)	.003		–3.34 (0.63)	<.001	
		Time spend x time point		–0.03 (0.01)	.02		–0.00 (0.00)	.35	

^a^PHQ-9: Patient Health Questionnaire.

^b^GAD-7: Generalized Anxiety questionnaire.

^c^Two-tailed *P* value.

^d^Pretreatment to last observation.

^e^*d* values cannot meaningfully be calculated for the interaction effects.

^f^FQ: Fear Questionnaire.

For patients with anxiety, a higher level of baseline anxiety predicted a slightly higher risk of noncompletion (OR=1.08, CI=1.01-1.16, *P*=.03). Similar results were noted for higher levels of comorbid depression (OR=1.12, CI=1.05-1.20, *P*<.001). A college education as the highest education level decreased the risk of noncompletion compared to primary school education (OR=0.14, CI=0.02-0.61, *P*=.02). A complete overview of all analyses of predictors for treatment completion is presented in Multimedia Appendix 1.

## Discussion

### Aim

The aim of the present study was to evaluate the clinical effect of guided iCBT for depression and anxiety in routine care by analyzing routine outcome measurements from the dedicated iCBT clinic Internetpsykiatrien in Odense, Denmark. From March 1, 2015, to June 1, 2017, 203 patients commenced treatment, including 60 patients who underwent treatment for depression and 143 patients who underwent treatment for anxiety, which demonstrates the need for and motivation to seek guided iCBT.

### Participants

Of the patients with depression, 78.3% were female, which corresponds to the findings of previous epidemiological research [49-52]. Despite the new format of treatment delivery, the gender difference may be biased by treatment-seeking behavior, as previously shown by a male to female ratio of 2:1. The prevalence of anxiety disorders is not extensively researched in Denmark, but in general practice, more women experience anxiety disorders than men [52]. The mean age, marital status, and employment status of our sample resembles those in primary care [52], which is reasonable, as Internetpsykiatrien offers selfreferral and is thus likely to receive a patient population of patients with depression and anxiety in primary care. The distribution of the highest education level resembles that of the general Danish population: primary, 25.9%; gymnasium, 10.2%; college or vocational school, 51.7%; university, and 9.5; other, 2.65% [53]. However, there are some noticeable differences. In the present study, fewer patients had primary school and university education as the highest level of education and more patients had college or vocational school as the highest level of education. Nonetheless, overall, our sample seemed representative of the Danish population.

### Primary Analyses

The main analyses of change in the symptom levels of primary disorder from pretreatment to posttreatment showed large and significant reductions in both depression (beta=–6.27, SE 0.83, *P*<.001, *d*=1.0), and anxiety (beta=–3.78, SE 0.43, *P*<.001, *d*=1.1). These results resemble within-group effect sizes of previous studies on guided iCBT for adult patients with anxiety and depression, including both efficacy studies conducted under controlled research conditions [54-56] and effectiveness studies conducted under routine care conditions [15]. Notably, the results of studies in clinics akin to the one under investigation, which employed similar study designs examining cohorts in routine care (*d*=0.9-1.4) [20-25], are in line with our results. Therefore, this study concludes that the clinic under investigation yields results similar to those seen in other comparable clinics, and the effects of iCBT are satisfactory as compared to within-group effect sizes of previous efficacy and effectiveness studies.

### Predictor Analyses

Baseline severity of primary disorders influenced the effect of treatment. For anxiety and depression, a higher baseline level of primary disorder correlated with larger treatment gains, which is a correlation commonly observed in studies on guided iCBT [57-59]. This effect is interesting, because it is often assumed that guided selfhelp should be aimed primarily at milder symptom levels of the disorders, although factors other than severity should be examined as indicators for stratification of patients for different treatments. Some studies found substantial evidence that treatment effect was unrelated to the treatment outcome [60].

There was a difference in how the symptomatic level of a comorbid disorder affected treatment gain. For patients with depression, comorbid anxiety slightly hampered the effect of treatment; in contrast, for patients with anxiety, high levels of comorbid depression increased the effect of treatment to a small extent. Thus, comorbidity might influence the effect of treatment differently for the two disorders, which may add nuance to the findings of a previous study, which concluded that the severity of comorbid disorders predicts poor treatment outcome [59]. Our finding may also support the use of transdiagnostic treatment programs or personalized treatment approaches.

For patients with anxiety, occupational status in the “Other” category rather than the “Employed/student” category positively affected the outcome (beta=–3.49, SE 1.10, *P*=.002). Analysis of the reasons for selection of the “Other” category showed that this category was extensively heterogeneous and included several entries such as apprenticeships, other forms of benefit, or no income, which made it difficult to interpret the result. Neither this category nor any other category of occupational status had an effect on the treatment in patients with depression. However, compared to the employed or student status, the unemployed status was associated with a higher risk of noncompletion for patients with depression. A previous study showed that a poorer employment status was associated with worse treatment outcome in unguided iCBT [61], which was not observed in this study. This difference in results may have resulted from the different formats of iCBT delivery. The present study investigated guided iCBT; therefore, the guidance may serve as a protective factor for vulnerable patients. This assumption could be substantiated by emerging findings that indicate the importance of the therapeutic alliance in iCBT [62,63]. Further study of the influences of and processes involved in the guidance is needed.

We found that more time spent in the program predicted a greater reduction in symptom severity for patients with depression but not for patients with anxiety, which seems to indicate a dose-response effect of depression treatment, but not anxiety treatment. Multivariate analyses showed different results for both anxiety and depression. For depression, no significant interactions were observed, which might be a result of the small sample size. These predictions should therefore be interpreted carefully.

### Completion

Of the patients who commenced treatment, 37 of 60 patients with depression and 57 of 143 patients with anxiety completed treatment, which yields noncompletion rates of 38.3% and 60.1%, respectively. A review of 152 studies on traditional face-to-face psychotherapy reported an average dropout rate of 46.9% [64], which is comparable to our result. However, the dropout rates of iCBT treatment usually range from 0% to 78%, with a weighted mean of 26.5% [65]. This finding is in line with the results from routine-care iCBT clinics in Sweden, Norway, and Canada, where rates of noncompletion ranged from 15.9% to 29.5% [66-68]. Therefore, in the Internetpsykiatrien clinic, the rate of noncompletion seems relatively high, particularly for patients with anxiety. However, it is possible that the cutoff chosen to signify completion of treatment in our study was overly conservative, requiring completion of 83% and 88% of the modules of depression and anxiety, respectively, which are considerably higher than the thresholds used in other studies [23], where merely 56% of the modules needed to be completed. This hypothesis may be substantiated by the fact that significant and moderate-to-large effect sizes were seen among patients who did not complete the programs in the present study, indicating that some patients may terminate their treatment because they are satisfied with their progress. In addition, the difference in effect size between intention-to-treat analyses and completer analyses was not significant. For both disorders, a higher baseline symptom level of comorbid disorders slightly increased the risk of noncompletion. Interestingly, the dropout rates differed between the two treatment groups, with higher dropout rates observed for patients with anxiety than for patients with depression (60.1% vs 38.3%). However, this does not appear to be the case in the literature [66-68], and the underlying reasons are unclear. This difference may be caused by technical variations such as those between programs or the format of delivery of support, for example, written support for depression treatment and telephone support for anxiety treatment. However, it could also be a result of the different symptomatology of the disorders; for example, anxiety is characterized by avoidant behavior. Nonetheless, it is important to determine how to improve adherence, particularly for patients with anxiety, and whether this goal can be attained by different methods for the two different disorders. Further studies should focus on examination of different user behavior and experiences from the programs during and after provision of support. Furthermore, examination of motivations for ending treatment is needed. Finally, research investigating process factors involved in completion or noncompletion is needed to further clarify individual differences in response patterns among patients receiving iCBT.

### Limitations

Most of the results from the exploratory predictor analyses were weak and subject to type 1 error due to their exploratory nature; therefore, they should be interpreted with caution. Prediction of treatment outcomes from baseline characteristics is generally difficult, as reported in previous iCBT studies showing varying results [58-61]. Our results therefore need repetition, particularly in routine care, with a larger study sample.

### Conclusion

The Internetpsykiatrien clinic has demonstrated its ability to implement iCBT in routine care as well as the existence of, need for, and motivation among patients to seek iCBT treatment for anxiety and depression in the Region of Southern Denmark. The clinic yields results comparable to those of similar international clinics and efficacy and effectiveness studies. As such, the Danish clinic shows good clinical effectiveness. These findings are important, because depression and anxiety are highly prevalent and costly disorders that are substantially undertreated. Our results support the hypothesis that iCBT can help bridge the gap between the need for treatment and its provision even in routine care. Further research is needed to investigate the processes of change and individual response patterns to iCBT in order to improve the ability to personalize treatment for the individual and understand the reasons for premature cessation of treatment.

## Appendix

Multimedia Appendix 1 Univariate logistic mixed-effects model interactions for prediction of noncompletion.

## Abbreviations

FQ: Fear Questionnaire
GAD-7: Generalized Anxiety Disorder-7
iCBT: Internet-based cognitive behavioral therapy
OR: odds ratio
PHQ-9: Patient Health Questionnaire-9
RCT: randomized controlled trial
