# Supplementary material for: Guided Internet-Based Cognitive Behavioral Therapy for Adult Depression and Anxiety in Routine Secondary Care: Observational Study
Source: J Med Internet Res. 2018 Nov 28;20(11):e10927. doi: 10.2196/10927 (PMC6291683; doi:10.2196/10927)
Supplement: Multimedia Appendix 1 [file jmir_v20i11e10927_app1.pdf]

Variable	Depression		Anxiety	
	Odds ratio (95% CI)	2-Tailed *P* Value	Odds ratio (95% CI)	2-Tailed *P* Value
Gender (male, female)	0.44 (0.12 - 1.54)	.200	0.86 (0.41 - 1.78)	.672
Age	0.96 (0.90 - 1.01)	.097	1.00 (0.98 - 1.03)	.902
Highest education level				
Gymnasium vs. Primary	7.00 (0.79 – 157.98)	.117	0.67 (0.09 – 3.24)	.642
College vs. Primary	0.64 (0.16 - 2.60)	.518	0.14 (0.02 – 0.61)	.018
University vs. Primary			0.33 (0.04 – 1.91)	.244
Other vs. Primary	0.60 (0.09 – 3.47)	.572	1.39 (0.17 – 8.49)	.729
Marital Status				
Married or De Facto Not Living Together vs. Single	4.80 (0.99 – 26.52)	.023	0.91 (0.34 – 2.42)	.843
Married or De Facto Living Together vs. Single	2.26 (0.67 – 8.45)	.057	0.32 (0.13 – 0.73)	.010
Occupational status				
Unemployed vs. Employed/Student	7.50 (1.40 – 59.25)	.028	1.44 (0.58 – 3.75)	.440
Sick Leave vs. Employed/Student	1.79 (0.42 – 7.41)	.420	0.96 (0.33 – 2.95)	.937
Other vs. Employed/Student	1.25 (0.27 – 5.27)	.764	0.43 (0.18 - 1.04)	.064
Baseline (Co)morbidity				
PHQ-9	1.00 (0.91 – 1.10)	.990	1.12 (1.05 - 1.20)	<.001
FQ total	1.03 (1.01 - 1.06)	.015		
GAD-7			1.08 (1.01 – 1.16)	.031
Psychotropic Medication				
<1 Month vs. No	0.00 (0.00 - 1.56e+122)	.992	2.95 (0.42 – 58.78)	.342
<2 Months vs. No Medication	3.00 (0.27 - 6.74)	.384	4.24e+06 (0.00 - ∞)	.988
>2 Months vs. No Medication	0.56 (0.11 - 2.43)	.438	1.99 (0.89 - 4.73)	.103
Units of Alcohol Weekly				
0-5 vs. 0	0.97 (0.33 - 2.84)	.962	0.89 (0.42 - 1.90)	.751
5-10 vs. 0	0.00 (0.00 - 1.80e+205)	.995	1.56 (0.52 – 5.36)	.448
10-20 vs. 0	0.00 (0.00 - 2.97e+108)	.992	1.00 (0.22 - 5.23)	1.000
>20 vs. 0			1.27e+06 (0-∞)	.987

PHQ-9: Patient Health Questionnaire-9,
FQ: Fear Questionnaire
GAD-7 Generalized Anxiety Disorder-7 Scale
